# Virtual Tele-Ultrasound in Pulmonary Ultrasound Peer-Education of Medical Students: A Preliminary Equivalence Study

**DOI:** 10.1016/j.acepjo.2025.100321

**Published:** 2026-01-22

**Authors:** Jennifer Wang, Ami Tamhaney, Torey Averick, Shreya Mathur, Thibault Philippine, Anna Jackanich, Charlene Gaw, Alan Chiem

**Affiliations:** 1 UCLA School of Medicine, Los Angeles, CA; 2 UCLA/Olive View Department of Emergency Medicine, Los Angeles, CA; 3 University of Michigan Emergency Medicine Residency, Ann Arbor, MI

**Keywords:** education, lung ultrasound, medical education, peer, pulmonary ultrasound, skills, teaching, teleguidance, training, ultrasonography, virtual

## Abstract

**Objectives:**

Peer-instructed tele-ultrasound has the potential to provide high-quality clinical ultrasound education to medical students. However, there is limited data evaluating the effectiveness of such methods in practice. The primary aim of this study is to evaluate how virtual tele-ultrasound teaching compares with traditional in-person teaching of peer-instructed pulmonary ultrasound in undergraduate medical students.

**Methods:**

In a preliminary single-center study, first-year medical students (*n* = 39) were randomized into 2 peer-instructed pulmonary ultrasound teaching groups: a traditional in-person group or a virtual tele-ultrasound group. Effectiveness was evaluated by 3 primary outcomes: (1) change in knowledge score on pre- and posttest, (2) performance in an objective structured clinical exam, and (3) subjective confidence surveys. The secondary outcome was participants’ overall experience with the teaching method. Two one-sided *t* test was used to measure equivalence between the 2 groups (*p* < .05).

**Results:**

The virtual teaching group was statistically equivalent to the traditional in-person group in all primary outcomes of knowledge change (37.4 vs. 37.8 point improvement out of 100, *p* < .001), OSCE score (12.7 vs. 12.4 out of 15, *p* = .002), and overall confidence (4.2 vs. 4.1 out of 5, *p* = .02). The tele-ultrasound group rated their experience highly overall, but not statistically equivalent to the traditional group (4.5 vs. 4.9 out of 5, *p* = .47).

**Conclusion:**

Peer-instructed tele-ultrasound may be an effective method of teaching pulmonary ultrasound to undergraduate medical students as compared with traditional in-person teaching.

**The Bottom Line**

Peer-instructed tele-ultrasound can potentially address the rising need for accessible lung ultrasound education; however, its effectiveness lacks substantial evaluation. This study randomized 39 medical students into tele-ultrasound and in-person teaching groups for pulmonary ultrasound training. The tele-ultrasound group performed equally well in knowledge change, clinical exam scores, and confidence, but rated their overall experience lower than the in-person group. This preliminary work shows that tele-ultrasound is a promising method of teaching pulmonary ultrasound knobology and image acquisition. Future work is needed to demonstrate generalizability, retention of skills, and applicability to real-world clinical situations.

## 1 Introduction

### 1.1 Background

Tele-ultrasound is a virtual ultrasound telemedicine platform that enables a remote practitioner to video call with an ultrasound user, visualizing both the user and the real-time ultrasound images. This allows the practitioner to provide real-time feedback on the user’s technique and image acquisition.^1^ This modality addresses the growing need for virtual ultrasound teaching methods as the need for pulmonary ultrasound education surged following the COVID pandemic and with the increasing format of the flipped classroom.^2^^,^^3^ Unfortunately, there is limited data on its application for teaching, especially in tandem with other highly used methods like peer-assisted learning (PAL).^4^^,^^5^ PAL allows students to learn from other students, providing cognitive and social congruence between learner and peer instructor and increasing opportunities for learning when faculty availability is limited.^6^, ^7^, ^8^ Prior studies integrating tele-ultrasound and PAL techniques in undergraduate medical education have proved success, such as the foundational work in Zhao et al^9^ which this study is built.^1^ However, there are limited studies evaluating broader application in other subjects such as lung ultrasound, which is expanding in clinical application.^10^

### 1.2 Importance

The COVID-19 pandemic highlighted alternatives to in-person learning and demonstrated the success of many such programs. Furthermore, it has showcased the clinical utility of lung ultrasound, particularly with the remote scanning capabilities of tele-ultrasound.^11^, ^12^, ^13^ However, there is limited data evaluating the effectiveness of such methods in educational practice.

### 1.3 Goals of This Investigation

The objective of this preliminary study was to evaluate how virtual tele-ultrasound teaching compares with traditional in-person teaching of peer-instructed pulmonary ultrasound of undergraduate medical learners. Specifically, we aimed to compare participants’ knowledge gain, image acquisition skills, and confidence between the 2 teaching style groups.

## 2 Methods

### 2.1 Study Design

**Figure 1:**
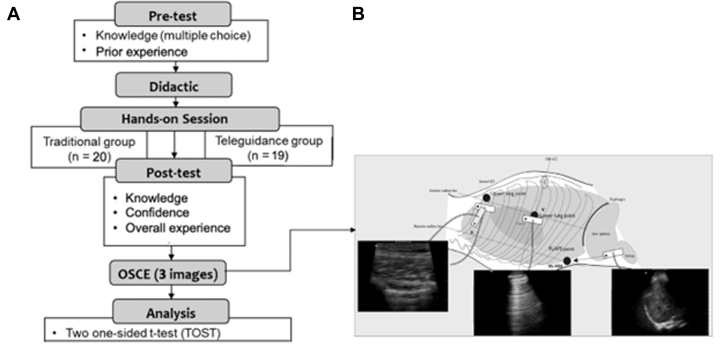
(A) (left) and (B) (right): Study design and OSCE lung exam. OSCE, objective structured clinical exam.

This was a randomized controlled equivalence study performed at a single California medical school comparing virtual tele-ultrasound teaching to traditional in-person teaching of peer-instructed pulmonary ultrasound to medical students. This study methodology was previously published with the same senior author in Zhao et al^9^ in application to ocular ultrasound teaching. This study applies the methodology to pulmonary ultrasound teaching. The study design is illustrated in Figure 1A. The participants were not the same as those in Zhao et al’s^9^ study. Primary outcomes were objective structured clinical exam (OSCE) performance, knowledge scores, and confidence scores. Secondary outcomes were overall experience and qualitative feedback from participants and instructors. These were chosen to provide a comprehensive assessment of the objective and subjective effectiveness of the interventions. This protocol was exempted by the institutional review board. All participants provided informed consent before participation.

### 2.2 Participants and Instructors

Participants were identified through a class email list. The eligibility criterion was being a first-year medical student having less than 1 year of medical ultrasound education. This was performed at a medical school that has a well-established longitudinal ultrasound curriculum, with approximately 8 hours of combined didactic and hands-on learning in the first-year class. Prior to enrollment, the goal sample size for a 2-sample equivalence test for 2 means was calculated (note these were estimated values, please see “Methods: Data Analysis” section for the implemented statistical methods). For this estimation, the type 1 error rate was set at 5%, the delta equivalence bound of 20%, the standard deviation of 20%, and the projected means of 30% (ie, the predicted change in knowledge scores of students before and after intervention, such as an improvement from 60% on pretest to 90% on posttest). Initial recruitment was performed via email and social media postings to the entire current first-year class of 175 students and was extended to the incoming first-year class of an additional 175 students to adequately power the study. Of the 350 eligible students, 66 students initially signed up to participate, and 39 participants ultimately attended the study. These 39 students were randomly assigned to 20 in the traditional in-person group and 19 in the virtual tele-ultrasound group. $20 gift cards were provided to participants.

Six peer instructors were selected from the medical student coordinators of the medical school’s Ultrasound Interest Group. These 6 peer instructors were peer medical students, 4 of whom were current first years, and 2 of whom were fourth years. All peer instructors underwent a standardized 2-hour training with an ultrasound fellowship-trained faculty member reviewing lung ultrasound principles, key teaching principles, and hands-on practice for instructors to teach with both in-person and virtual tele-ultrasound modalities using volunteer models. A standardized list of key teaching points was given to instructors during teaching sessions to promote content consistency between instructors. The same faculty member and first author monitored the sessions and supervised the teaching. The same instructors were used to teach both study groups. An equal number of first-year and fourth-year peer instructors were represented in each study intervention group.

### 2.3 Materials and Setup

The tele-ultrasound platform used was Butterfly Inc’s Teleguidance system (Butterfly Inc, https://www.butterflynetwork.com/teleguidance) using Butterfly’s portable iQ+ probes (https://www.butterflynetwork.com/iq-plus) that connected to iPads (Apple) with the Butterfly iQ+ application. These devices were rented from the school’s simulation center, in addition to ultrasound gel and towels. Butterfly iQ+ accounts and cloud image storage were provided by Butterfly Inc.

Sessions were held in reserved medical school classrooms. Each tele-ultrasound group had a Butterfly iQ+ probe connected to an iPad with the Butterfly application, and their remote instructors called in using tele-ultrasound from their personal laptops in a separate room. In-person instructors were present in the same room as participants. Participants of both groups acted as peer models and alternated scanning each other. If none of the participants in the group was able to peer model, an extra peer instructor or investigator filled in as the model.

### 2.4 Intervention

The intervention was either a virtual tele-ultrasound or an in-person teaching session with the peer instructors. Participants were given an anonymized participant ID number and randomized into either group using a random group generator (https://www.randomlists.com/team-generator), performed concealed with a 1:1 allocation ratio. Within each intervention type, participants were divided into small groups of 2 to 4 participants and 1 peer instructor. Then, a peer instructor gave a brief, 7-minute orientation and didactic on pulmonary ultrasound basics to the whole group. This introduction time was made brief so that the bulk of teaching was performed in the divided study arms. Participants were then randomized into small groups in separate rooms with either an in-person or virtual tele-ultrasound peer instructor. Both groups were allowed 45 minutes of instruction and practice time. Each instructor taught from a checklist of standardized teaching points, which included knobology, probe movements, gain, depth, identifying pertinent anatomy, and saving an image of the view. The teaching topic was pulmonary ultrasound based on the BLUE-protocol.^10^ Teaching and assessment objectives focused on learning goals for preclinical learners with limited ultrasound experience, with a primary focus on technique and image acquisition. Instructors were told to evenly distribute the amount of hands-on practice among participants by monitoring time and allocating approximately the same amount to each learner.

### 2.5 Evaluation

Prior to the teaching session, participants completed an anonymized survey evaluating hours of prior ultrasound experience and a knowledge pretest to evaluate their baseline characteristics. The prior experience survey assessed participants’ hours of prior ultrasound experience and lung-specific ultrasound experience. The knowledge pretest consisted of 9 multiple-choice questions that focused on key lung ultrasound concepts delivered through the skill session.

Ten minutes after the teaching session, each student took an anonymized knowledge and confidence posttest with questions identical to the pretest. This posttest was 1 hour after the pretest. Students also completed a survey on their overall experience in either the traditional or tele-ultrasound teaching group and their confidence in their skills. Both surveys used a 5-point Likert scale and were administered via the students’ mobile devices without the peer instructor present in the room. All tests and surveys were anonymized using participant ID numbers and created using Qualtrics.

After the posttest, students took an OSCE on pulmonary ultrasound image acquisition. All students were given written instructions to save 3 ultrasound images: (1) identify right upper anterior lung sliding, (2) evaluate for A-lines and B-lines at the right lateral point, and (3) evaluate for right posterior pleural effusion by looking for the “spine sign” view (Fig. 1B). The instructions stated to use the preset, gain, and depth that they thought would be best for image acquisition. The peer instructors served as patient models but did not give any additional verbal instruction to the students. Images were saved to a cloud server and annotated with the participant’s anonymous ID number for asynchronous review.

### 2.6 Measurements

**Figure 2:**
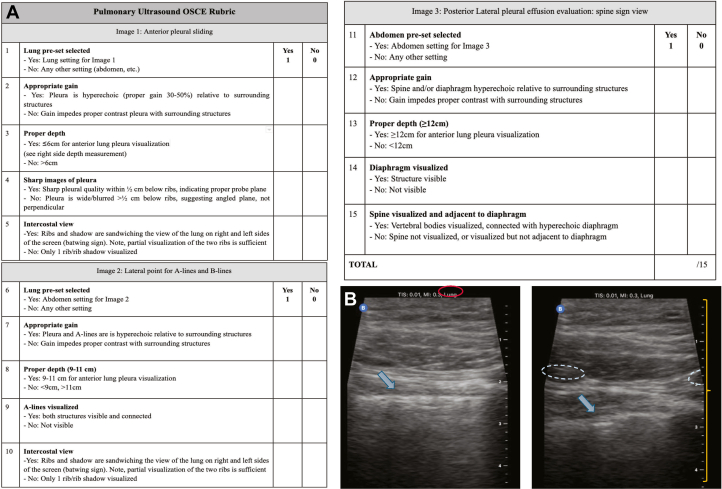
(A) OSCE scoring rubric. (B) Sample image 1s from OSCE. The left image demonstrates sharp hyperechoic pleura, whereas the right image shows blurred pleura (arrows). Both are in the appropriate lung preset (circled at the top left), depth (centimeter depth marked on the right), and intercostal view (ribs circled with dashed marks on the right). OSCE, objective structured clinical exam.

The 3 images obtained during the OSCE were scored based on a set rubric (Fig. 2A). Images were awarded 1 point for each criterion that was met, with a maximum of 5 points per image for a total of 15 points (example of image 1 in Fig. 2B). Each image was scored by 2 designated reviewers who were blinded to whom the participant was and which intervention group they were part of. These reviewers were senior emergency medicine residents from the medical school’s affiliated residency program, trained via the residency’s internal ultrasound program by ultrasound fellowship-accredited physicians. Reviewers were oriented by the primary author and given a standardized rubric to score from with sample images to reference. The 2 reviewers independently scored each OSCE image, and the scores of the 2 reviewers were averaged to generate the final score. An inter-rater reliability was calculated to evaluate the degree of agreement of reviewers.

### 2.7 Data Analysis

The outcomes of the virtual tele-ultrasound groups and traditional in-person groups were analyzed using the 2-sample *t* test equivalence testing for continuous, normally distributed variables (knowledge score, OSCE score, confidence score). Equivalence testing was used based on Lakens et al^14^ and Zhao et al^9^ in which the equivalence bound was the smallest effect size of interest (SESOI) based on the maximal clinically acceptable difference.^15^ The investigators established this SESOI to be 1 standard deviation of the control group because this was deemed a clinically important difference in novice preclinical learners based on the investigators’ experience in ultrasound education (ie, if 2 students scored within 1 standard deviation of each other, this was deemed equivalent performance). This was applied to all outcomes of the knowledge test, OSCE, experience, and confidence. Analysis was performed in Excel (Microsoft), and figures were plotted with Excel and R.

The distribution of outcomes was examined to determine the appropriateness of parametric vs. nonparametric testing in our smaller sample size. Knowledge change (skewness = 0.10) and confidence scores (skewness = 0.0) were normally distributed, whereas OSCE score (skewness = −1.1) and experience score (skewness = −0.60) were moderately negatively skewed. Wilcoxon-Mann-Whitney’s two one-sided *t* test (TOST) for between-group symmetry in equivalence bounds was performed as described in Caldwell^16^ showing that nonparametric equivalence tests concluded the same equivalence findings as those from the parametric TOSTs (*p* < .05) with parametric testing being more highly powered. After iterative discussion with statistical consultants, parametric testing was felt most appropriate for this study.

## 3 Results

**Figure 3:**
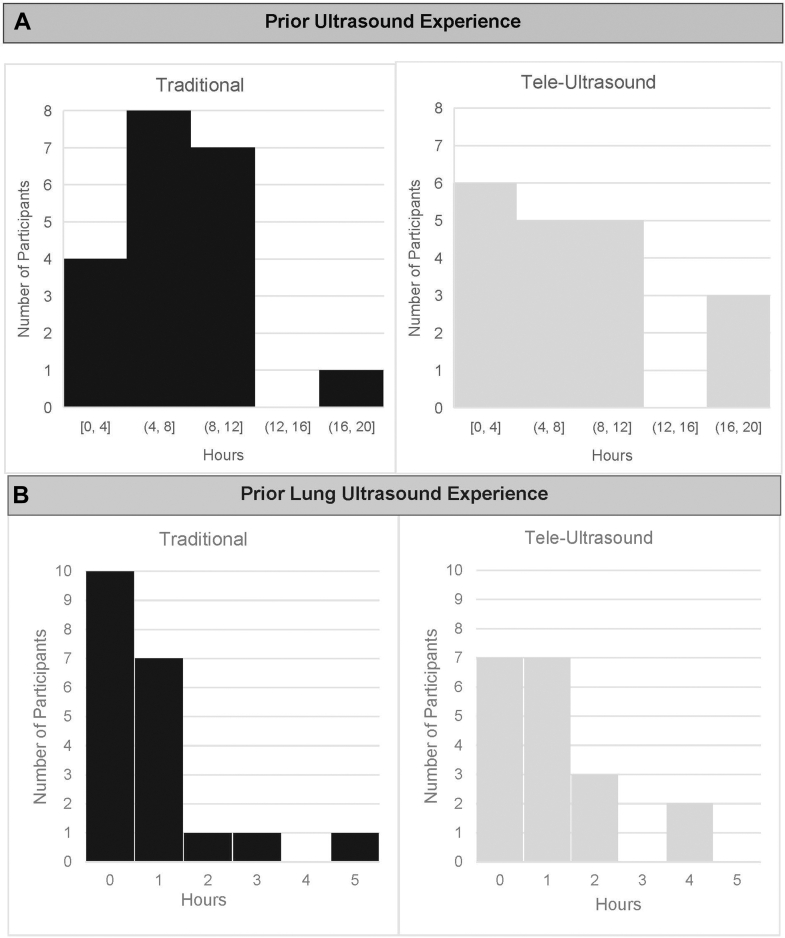
(A) and (B) Participants’ prior ultrasound experience.

**Figure 4:**
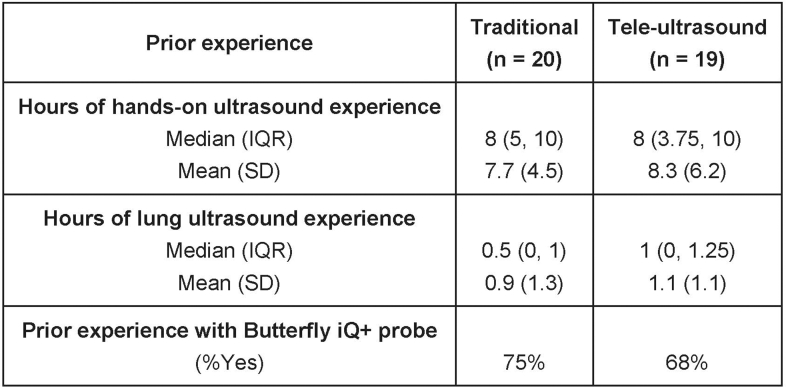
Participant characteristics.

The study enrolled 39 participants: 20 in the in-person group and 19 in the tele-ultrasound group. The baseline characteristics for the traditional in-person group and virtual tele-ultrasound group were as follows: the median prior hours of ultrasound experience was 8 (IQR of 5-10) and 8 (IQR 3.75-10), respectively, and the median hours of lung-specific ultrasound experience was 0.5 (IQR 0-1) and 1 (IQR 0-1.25), respectively (Fig. 3A, B). In the traditional in-person group, 75% had used the Butterfly iQ+ probe before, and in the virtual tele-ultrasound group, 68% had used it before (Fig. 4).

**Figure 5:**
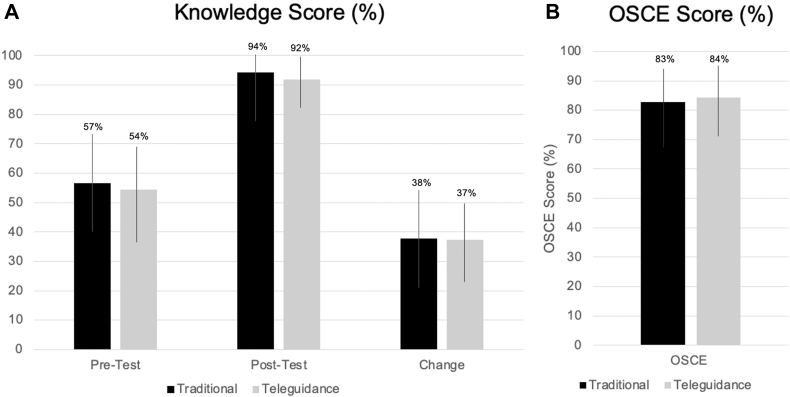
(A) Knowledge and OSCE score table. (B) Knowledge and OSCE score graph. OSCE, objective structured clinical exam.

The tele-ultrasound group was statistically equivalent to the in-person group in all primary outcomes of knowledge change, OSCE score, and overall confidence as demonstrated in Figures 5a/b and 6. The confidence and subjective experience score are shown in Figure 7. The numerical equivalence bounds represent the predefined range of differences (±SESOI) within which the 2 groups would be considered educationally and practically equivalent. A significant p value in the TOST analysis indicates that the observed difference lies entirely within these bounds, demonstrating statistical equivalence. The inter-rater reliability score for OSCE scoring was 0.6, suggesting moderate to substantial agreement among reviewers. For the subjective survey of overall experience, tele-ultrasound was nonequivalent to the traditional group but still rated highly at 4.5 compared with 4.9, respectively (p = .47). Participants reported occasional technical challenges with the volume of tele-ultrasound being too quiet or difficulties setting up equipment and troubleshooting camera positioning on their own. Tele-ultrasound instructors reported overall positive experiences with the tele-ultrasound modality and were more comfortable after the training session and the first teaching session.

**Figure 6:**
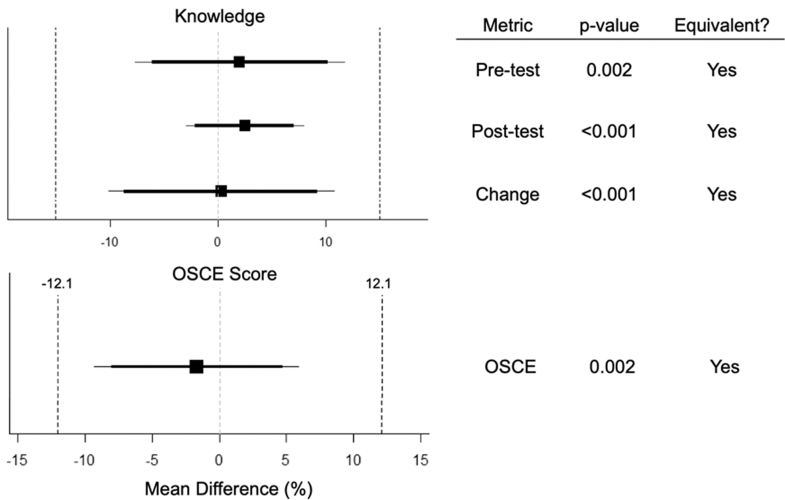
Knowledge and OSCE score equivalence plots. OSCE, objective structured clinical exam.

**Figure 7:**
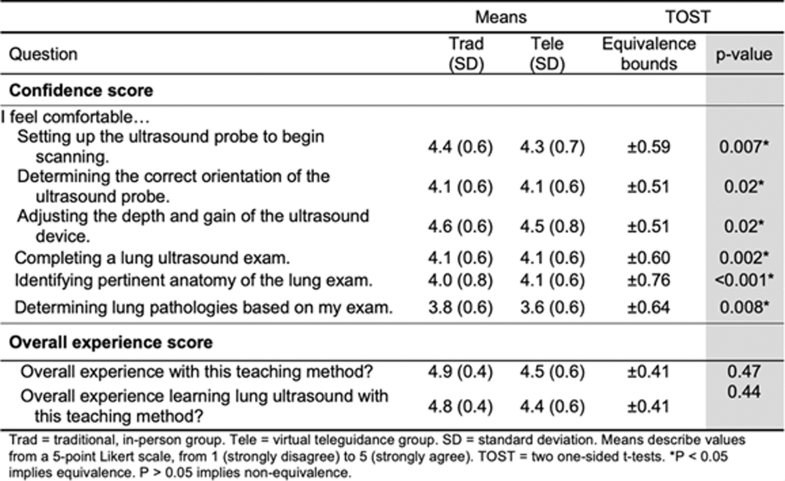
Confidence and experience score equivalence results.

### 3.1 Limitations

This was a follow-up of a prior pilot study performed on ocular ultrasound by our research group that replicated similar findings in pulmonary ultrasound. The primary limitation of this study was its small sample size and limited generalizability since only 39 of the eligible 350 students elected to participate. This introduces selection bias in our participant group because this was a voluntary extracurricular activity. Those participating may have increased baseline knowledge, experience, and motivation in ultrasound, making it more likely that the 2 experiment groups were equivalent. In anticipation of this, the presurvey and pretest were used to characterize the study population’s baseline knowledge and experience so outcomes could focus on change in knowledge rather than baseline knowledge. Future studies should explore how these results are affected by this being a required rather than an optional activity.

Another limitation is that the assessments were conducted promptly after learning and may not reflect long-term knowledge gain. The posttest was performed about 1 hour following the pretest, which could lead to recollection bias. Additionally, both groups were taught by the same instructors by design to try and reduce the variability of different instructors’ effectiveness; however, this could introduce bias from a lack of blinding and make it more likely that the 2 groups would be equivalent. Although intergroup variability was minimized through the randomization protocol and standardized teaching methods, there was still variance in group size, time of individual teaching, and instructor experience.

There are inherent limitations to using live models as our OSCE subjects including differences in thoracic anatomy, gender, and body habitus. We initially considered using a lung ultrasound simulation trainer for testing to standardize the testing experience; however, these were unavailable and cost-prohibitive to acquire. Additionally, our live models had normal physiology, so recognition of abnormal pathologies was not assessed in the OSCE. The OSCE primarily assessed knobology and image acquisition.

There was also variability among image scorings, although ultimately moderate inter-rater reliability of 0.6. Further training or additional observers may help improve upon this result. For example, creating a certification set of images to calibrate the scores or having a third-party reviewer to resolve discrepancies over a certain score difference threshold. Senior resident reviewers were chosen based on their competency in lung ultrasound and availability; however, in the future, faculty reviewers could be considered if resources allowed.

There is a lack of consensus on how to determine the SESOI for equivalence. Some may suggest an SESOI of 1 standard deviation is too large because some equivalence study methodologies propose a *d* = 0.5 as a moderate effect and *d* = 0.8 as a large effect size. However, we again emphasize that the principle for determining SESOI is based on what is a clinically acceptable maximal difference. Clinically, based on our expert-derived opinion and prior studies in the field, this was determined to be 1 standard deviation for novice preclinical learners.^9^^,^^15^ Future work should standardize and validate these analysis methods.

## 4 Discussion

Tele-ultrasound is a promising method of teaching medical students in a broad set of applications, settings, and instruction methods.^4^^,^^5^^,^^17^ This preliminary pilot study demonstrates the potential of using tele-ultrasound and PAL together to teach undergraduate medical students in lung ultrasound, expanding the ways that tele-ultrasound can be used. The tele-ultrasound group’s equivalent performance as the traditional group in all primary aptitude outcomes suggests this may be an effective comparable modality, although additional studies with larger sample sizes are needed. The nonequivalent overall experience score and subjective feedback highlight areas for future improvement.

Overall, the tele-ultrasound group rated the experience highly but not statistically equivalent to the traditional group. Previous studies support this finding that tele-ultrasound students perform just as well, but often still prefer in-person traditional teaching.^4^ Based on subjective feedback from participants, this could be due to technical challenges like difficulty hearing the tele-ultrasound instructor, iPad positioning for concurrent user and instructor viewing, or independently troubleshooting the application. This could be mitigated by technical testing beforehand, iPad stands with pre-optimized camera positioning, and clear troubleshooting instructions. Prior studies have also shown that personal characteristics such as age, comfort with technology, and attitudes toward technology also have significant impacts on how students perceive remote learning.^18^ Additionally, informing students of the evidence showing equivalent learning outcomes may help assure them of their confidence in this modality.^4^^,^^5^^,^^19^

From this study process, we learned that recruitment and retention were key challenges. Of the 350 eligible medical students, only 66 signed up (19% of eligible participants), and of those, only 39 were able to attend (11% of eligible participants). Because this was an optional extracurricular activity after class rather than an integrated study in the existing curriculum, it was challenging for medical students with limited time to stay for an additional 2 hours for this study. We hope our preliminary findings can pave the way for future curricular implementation, which would facilitate future work from a recruitment and retention perspective. Future studies with larger samples are needed to consider tele-ultrasound and in-person teaching as equally effective. Future work could consider evaluating long-term retention, comparing PAL tele-ultrasound with traditional non-PAL faculty-led instruction, and curricular implementation.^20^

In conclusion, this preliminary study shows the potential promise of peer-instructed tele-ultrasound for pulmonary ultrasound teaching of medical students. There is a broad application in expanding accessibility in ultrasound education, and it addresses the increasing need for effective lung ultrasound training.^12^^,^^21^ This is an early pilot study, and further studies in tele-ultrasound application are needed to further demonstrate effectiveness and generalizability.

## Author Contributions

JW was the primary author, conceived and designed the analysis, collected the data, and performed the analysis.

AT, TA, SM, and TP have equal contributions in creating and conducting teaching methods, collecting data, and reviewing papers.

AJ and CG have equal contributions in teaching peer instructors, collecting data, and reviewing papers.

AC was the senior research supervisor; oversaw conception of design, data collection, and analysis; and edited the paper.

## Funding and Support

UCLA/Olive View Department of Emergency Medicine Research Department

## Conflict of Interest

All authors have affirmed they have no conflicts of interest to declare.
